# Regulation of Craving: Neural Correlates and the Role of Stress and Delay Discounting

**DOI:** 10.1111/adb.70190

**Published:** 2026-09-24

**Authors:** Solvej Nickel‐Grenke, Raoul Wüllhorst, Stefan Scherbaum, Kristina Schwarz, Tanja Endrass

**Affiliations:** ^1^ Dresden University of Technology Faculty of Psychology Dresden Germany

**Keywords:** addiction, delay discounting, fMRI, regulation of craving, smoking, stress

## Abstract

Regulation of craving (ROC) by focusing on long‐term negative consequences of drug use has been shown to successfully reduce subjective craving. This process is supported by the dorsolateral prefrontal cortex (dlPFC), which exerts control over reward and value signals in the ventral striatum (VS) and ventromedial prefrontal cortex (vmPFC). As stress impairs cognitive control and addiction is characterized by altered value computations manifesting in delay discounting, we examined how these factors interact with ROC and its neural correlates to better understand the use of ROC in high‐risk situations. Sixty‐eight male smokers underwent a delay discounting task and either a stress (socially evaluated cold pressor task) or a control condition. They subsequently completed a ROC task during fMRI while focusing on (1) long‐term negative consequences (LATER) versus (2) short‐term positive consequences (NOW). We found lower subjective craving ratings and attenuated neural responses in the VS (*t* = 4.33, *p* = 0.012) and vmPFC (*t* = 5.79, *p* < 0.001), as well as higher activation in the dlPFC (*t* = 4.09, *p* = 0.038) in the LATER compared to the NOW strategy. Although no neural or behavioural difference in ROC was observed between stress and control groups, both individual stress (cortisol) and delay discounting were positively related to vmPFC activity (NOW > LATER). We replicated previous results on ROC. Importantly, the association of vmPFC signals with stress response and delay discounting suggests that enhancing ROC success may involve reinforcing changes in self‐referentiality of future thoughts to subjective value of drug use.

## Graphical abstract

Sixty‐eight male smokers underwent delay discounting and stress/control conditions before completing a regulation of craving (ROC) task during fMRI. Focusing on long‐term negative consequences was associated with reduced subjective craving and lower ventral striatum and vmPFC activity, as well as higher dlPFC activation, compared with focusing on short‐term positive consequences, with no differences between stress and control groups. Individual stress response and delay discounting were positively associated with vmPFC activity, suggesting a role for future‐oriented self‐referential processing in ROC.

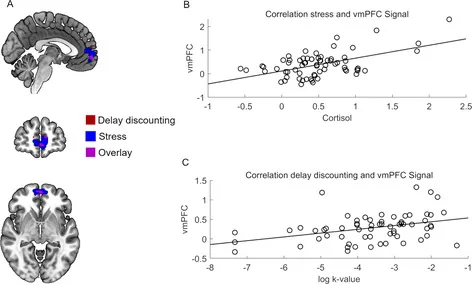

## 1 Background

Craving significantly influences substance use and relapse in individuals with substance use disorders (SUD [1]). It is defined as a strong desire or urge to use a substance and is part of the diagnostic criteria for SUD [2]. Craving is associated with brain activation in the ventral striatum (VS), ventromedial prefrontal cortex (vmPFC) and subgenual anterior cingulate cortex (sgACC [3]), which are thought to mediate reward processing and subjective decision values [4]. Craving can be elicited by cues associated with substance use, such as images of the substance [1]. When consistently linked to the time of use, these cues begin to predict the drug's reward, obtaining incentive salience and leading to craving and consumption even after the drug has lost its rewarding properties [5]. Accordingly, the primary motive for daily smokers to smoke is the need to reduce craving, accounting for 63% of smoking occasions [6]. It is therefore important for successful smoking cessation to reduce cue‐induced craving.

In situations where substance‐related cues cannot be avoided, cognitive regulation strategies are useful to reduce craving [7]. To investigate this in the laboratory, Kober et al. [8] developed the regulation of craving (ROC) paradigm. This approach has shown that focusing on the negative long‐term consequences (LATER) rather than on the short‐term positive effects (NOW) of substance use can effectively reduce cue‐induced craving across substances [8, 9, 10, 11]. Successful ROC is associated with attenuated activation in the VS/sgACC and vmPFC and with higher activation in frontal brain regions, such as the dorsolateral prefrontal cortex (dlPFC) and ventrolateral prefrontal cortex (vlPFC) [8, 11, 12].

Although the effectiveness of ROC under controlled laboratory conditions is well established, there is limited research on the factors that influence ROC success. Studies have shown that stress can significantly contribute to the occurrence of craving and relapse in SUD following periods of abstinence [13]. It has been demonstrated that coping with negative internal states such as stress is a pivotal motive for smoking [14] and associated with relapse [15]. Stress is defined as a threatening state due to potential adverse physical or psychological consequences characterized by perceived uncontrollability and unpredictability [16]. At the physiological level, stress activates the hypothalamus–pituitary–adrenal (HPA) axis [16], leading to the release of cortisol, which can be assessed through saliva samples. Stress‐related deficits are assumed to arise because cognitive resources are diverted to manage the stressor [17]. For example, cortisol released during stress is associated with impaired working memory [18]. Importantly, stress is hypothesized to impede prefrontal brain functioning [19]—regions that are pivotal for successful ROC [8]. Given that stress can induce or amplify craving [13], regulating craving may be both particularly important and challenging under stress. Thus, the first aim of the present study was to examine ROC and its neural correlates in smokers following stress exposure.

In addition to stress, personal beliefs about the occurrence of negative consequences may influence ROC success. For instance, ROC was more successful in individuals reporting higher future negative alcohol expectancies [11], and the anticipation of future negative physiological states was related to successful ROC [20]. This suggests that future‐oriented thinking, including subjective evaluations of expected consequences, is relevant to successfully implementing ROC. Previous research on the evaluation of consequences has shown that the frequency and extent of drug use and the severity of dependence were associated with a devaluation of reward when it was delayed [21]—a construct known as delay discounting (DD). As ROC focuses on delayed consequences, it is possible that the tendency to devalue future events also influences the effectiveness of ROC. Therefore, our second research question was whether ROC success was affected by DD.

To investigate how stress and DD affect ROC, daily smokers undergoing short‐term abstinence were randomly assigned to either a stress or a control group and performed an fMRI version of the ROC paradigm. This involved focusing on the long‐term negative (LATER) or short‐term positive (NOW) consequences of smoking or eating unhealthy food while viewing smoking‐ or food‐related images, respectively [8]. This was tested for both the ROC for cigarettes and food, as there is evidence that smokers respond less to naturally appetitive reinforcers [22]. Based on previous research [8, 11], we expected that the LATER compared to NOW strategy would yield lower subjective craving (i.e., ROC success) accompanied by lower brain activation in areas associated with craving (VS/sgACC, vmPFC/OFC) and higher activation in areas associated with cognitive control (dlPFC, vlPFC). Importantly, we hypothesized that stress would impair ROC success, resulting in reduced attenuation of subjective craving, lower activity in dlPFC and vlPFC and reduced attenuation of VS/sgACC and vmPFC/OFC activity in the stress group. We expected ROC success to correlate with lower DD, that is, a greater willingness to forgo immediate in favour of larger, delayed rewards.

## 2 Methods

### 2.1 Sample

This project was preregistered prior to data analyses (see https://osf.io/ydnzj/). We recruited *N* = 88 male smokers for the current study. Exclusions due to lack of data or quality (*n* = 20) resulted in a final sample of *n* = 68 (see Section S1.1.1 for information about sample size estimation and Section S1.1.2 for exclusion reasons).

Research staff conducted a telephone interview and assessed the following criteria (see Section S1.1.3 for exclusion criteria). Inclusion criteria were (1) smoking at least eight cigarettes per day for at least 1 year; (2) fulfilling at least two DSM‐5 criteria of tobacco use disorder (assessed with screening questions based on Structured Clinical Interview for DSM‐5 Clinical Version [SCID‐5‐CV] [23]); (3) fluency in German; (4) normal or corrected‐to‐normal vision; and (5) male gender, the latter ensured power regarding physiological responsivity to the stressor, which can be reduced in women compared to men [24] and smokers compared to non‐smokers [25]. Participants were randomly assigned to either stress (SG) or control group (CG; see Table 1 for sample characteristics) and provided informed consent in accordance with the Declaration of Helsinki. The local ethics committee approved the study protocol (reference number EK 53022018). Participants were compensated with 10 EUR per hour.

**TABLE 1:** Sample characteristics.

Variable	SG (*n* = 33)		CG (*n* = 35)		*t* (df)	*p*
Age	28.0	6.7	26.4	6.3	1.00 (66)	0.322
FTND	2.6	1.4	3.8	1.6	−3.36 (66)	0.002
DSM criteria	5.4	1.5	5.2	1.8	0.54 (66)	0.589
Cigarettes per day	11.9	3.7	12.6	4.1	−0.70 (66)	0.489
Abstinence cigarettes (h)	5.3	4.5	4.8	3.8	0.43 (66)	0.668
Abstinence food (h)	5.3	4.5	5.1	4.8	1.16 (66)	0.250

*Note:* Means and standard deviations are reported.

Abbreviations: CG, control group; DSM criteria, number of criteria met; FTND, Fagerström Test for Nicotine Dependence [26]; SG, stress group; *t*, test statistic.

### 2.2 Procedure

Participants attended two separate sessions scheduled on different days. The first session involved behavioural testing (~1 h) and assessed the DD task (see Section 2.5; see Sections S1.2 and S3.3 for further variables). The second session involved fMRI scanning during the ROC task and took place within 1 week after the first session (~2.5 h). Participants were asked to abstain from smoking and consuming food 3 h before the second session to ensure craving for cigarettes and food during the session. They underwent a Smokerlyzer breath test to determine carbon monoxide levels and were informed of this test during the first session to increase compliance with the abstinence requirement. Additionally, participants were asked to abstain from consuming alcohol for at least 24 h and caffeine for at least 1 h as well as sleeping for 1 h prior to testing as per recommendations for salivary cortisol assessments [27]. Participants first completed questionnaires before being introduced to the ROC task (see Section 2.3). Then, a baseline saliva sample (T1) was collected to measure cortisol immediately before the stress induction or control condition (see Section 2.4). A second saliva sample (T2) and a subjective aversiveness rating (‘How did you find the task with the water bucket?’ on a scale from 1 [*very pleasant*] to 5 [*very unpleasant*]) were collected in the scanner immediately before the ROC task commenced (~18 min after stress induction). A third saliva sample (T3) was obtained after completion of the task outside the scanner (~50 min after baseline).

### 2.3 Regulation of Craving Paradigm: ROC‐Task

**FIGURE 1:**
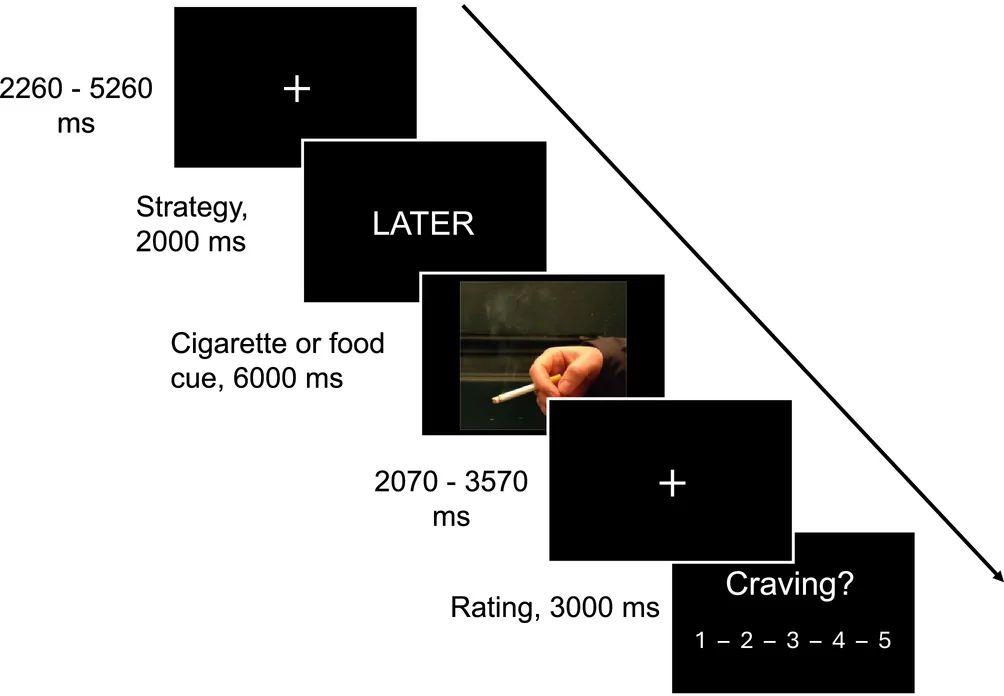
Regulation of craving (ROC) paradigm. *Note:* Task and trial timing. Scheme of one exemplary trial (showing a LATER trial). Trials were separated using a jittered intertrial interval of 2*1880 (repetition time) +/− 1500, 750 or 0 ms (*M* = 3760 ms). A jittered interstimulus interval of 1.5*1880 (repetition time) +/− 750, 375 or 0 ms (*M* = 2820 ms) was used between cue presentation and rating of craving. To prevent copyright issues, the image shown here was created to closely resemble stimulus content presented during the task.

The ROC task was scheduled to coincide with the expected peak in cortisol release [28]. During the task, participants were presented with cues either depicting cigarettes or high‐caloric food items [8]. Cigarette cues were partly taken from the Geneva Smoking Pictures database [29], and sets provided by the authors of prior cue reactivity studies [30, 31]. Food cues were taken from the FoodPics database [32]. Before each cue, a strategy instruction informed participants to imagine either the short‐term positive (NOW) or long‐term negative (LATER) consequences of repeatedly consuming the depicted item. After cue presentation, subjects indicated how strong their craving was while viewing the cue on a 5‐point Likert scale (1 = *none at all* to 5 = *very strong*). More details regarding timings are provided in Figure 1. A total of 50 food item cues and 50 cigarette cues (25 trials for each strategy type; 100 trials in total) were used. Trials were presented in pseudorandomized order with the constraint that only two consecutive trials of the same type could occur.

Participants conducted a practice block, during which they were asked to describe their subjective immediate positive and commonly known long‐term negative consequences of smoking and eating unhealthy food. The experimenter additionally mentioned any medical complications that were not mentioned from a list.

### 2.4 Stress Induction: Socially Evaluated Cold Pressor Test (SECPT)

We used a socially evaluated version of the cold pressor test (SECPT [33]), which has been shown to activate the sympathetic nervous system and the HPA axis as well as increase subjective stress levels. Participants immersed their non‐dominant hand and forearm in a bucket of water for 3 min. For the stress group (SG), the water was cold and included ice cubes (1°C–5°C), whereas for the control group (CG) the water was warm (30°C–35°C). In the SG, participants were not informed about the duration of the test and told that they were closely monitored for signs of pain or distress by the experimenter and via videotape from a camera placed in front of them. Participants were debriefed after the experiment that this was not true. In the CG, there was no recording or observation, and participants knew the duration in advance.

### 2.5 DD (Intertemporal Choice Task)

DD of rewards, measured as *k*‐value of a hyperbolic function [34], describes the tendency to prefer temporally proximate over more distal rewards, even if the former are smaller and the latter are larger. Based on Kräplin et al. [35], participants completed 192 trials without a response deadline. On each trial, they indicated whether they would hypothetically choose a smaller (randomly selected from a pool of items with M = €20, SD = €2 and range €17–€23) monetary amount delivered sooner (now, in 7, in 14 days) or a larger (increasing the smaller value by 1%, 3.5%, 7%, 12%, 18%, 27%, 40% or 80%) monetary amount delivered later (additional delay of 1, 2, 3, 5, 7, 9, 12 or 15 days).

### 2.6 Behavioural Analysis

Analyses were carried out using Matlab R2022b and JASP 0.16.2.

We have combined the analyses of food and smoking cues to increase the statistical power, but we also present a differentiated analysis of both types of cues.

#### 2.6.1 Task Effect

Subjective craving ratings of NOW and LATER trials were compared in the CG using a dependent samples *t*‐test. ROC success was quantified by subtracting mean craving ratings in the LATER strategy from mean craving ratings in the NOW strategy.

#### 2.6.2 Stress

As a manipulation test for the stress induction, we compared the aversiveness rating of the SECPT between groups using an independent samples *t*‐test. Regarding cortisol, we transformed cortisol readings according to *Χ'* = (*Χ* ^0.26^ – 1)/0.26, following recommendations by Miller and Plessow [36]. We computed reactivity scores by separately subtracting T1 and T3 from T2. We used independent samples *t*‐tests to compare groups at baseline (T1) and after the task (T3), as well as regarding reactivity scores. Craving ratings from the ROC task were subjected to a mixed‐model ANOVA using the between‐subjects factor group (CG, SG) and the within‐subjects factor strategy (NOW, LATER). We corrected the significance threshold α = 0.05 using Bonferroni–Holm and inequality of variance (tested with Levene's test) within the ttest2 function in Matlab. Next, we examined stress effects dimensionally as a function of the individual stress response. Therefore, in regression analyses across the entire sample, the aversiveness rating of the SECPT and individual cortisol reactivity scores (T2–T3) served as separate predictors of ROC success.

#### 2.6.3 DD

We estimated *k‐*values according to Kirby et al. [37] with higher values indicating that immediate rewards are overvalued. Due to incorrect key use, *k*‐values could not be determined for *n* = 5 participants, whom we excluded from the corresponding analyses. We log‐transformed DD *k*‐values to offset right skewness. To investigate the relationship between DD and ROC success, we set up a linear regression with ROC success as the dependent variable and the DD *k*‐value as regressor.

#### 2.6.4 Cue‐Type Effects

To examine the effect of cue‐type on subjective craving, we used a mixed‐model ANOVA with the factors group (SG, CG), strategy (NOW, LATER) and cue‐type (food, cigarette).

### 2.7 fMRI Acquisition and Data Analysis

Neural data were acquired using a 3‐T Siemens scanner, with a structural scan lasting ~7 min and a functional scan lasting ~32 min (for details, see Section S1.3) and submitted to standard preprocessing using SPM12 (for details, see Section S2.1). At the first level, regressors (NOW‐cigarette, LATER‐cigarette, NOW‐food, LATER‐food) were entered into a general linear model (GLM) together with regressors for the instruction, rating periods and six motion parameters as nuisance regressors. All task regressors were convolved with a canonical haemodynamic response function, and a high‐pass filter was applied to remove low‐frequency signal drifts. We computed the following first‐level t‐contrasts: NOW > LATER and LATER > NOW. To examine the effect of task at the neural level, we first subjected first‐level t‐contrasts (NOW > LATER and LATER > NOW) within the CG to second‐level one‐sample *t*‐tests.

We defined regions of interest (ROIs) based on previous ROC findings in smokers [8, 11]: We used spherical ROIs in VS/sgACC (−3, 11, −2; 6 mm radius) and vmPFC/OFC (0, 56, −2; 15 mm radius) for the craving circuit and around dlPFC (−36, −1, 55; 15 mm radius) and vlPFC (−45, 23, −8; 15 mm radius) for the control network. We transformed the coordinates into MNI space. Significance was assessed at the voxel‐level and defined as *p* < 0.005, familywise error (FWE) corrected within the ROIs. Outside these masks, voxel‐level significance was defined as *p*_FWE_ < 0.05, corrected across the whole brain. We conducted all analyses both at the whole brain level and within the ROIs (craving circuit for NOW > LATER, control network for LATER > NOW).

#### 2.7.1 Stress

We compared SG and CG in NOW > LATER and LATER > NOW contrasts using independent samples *t*‐tests. To examine stress dimensionally as a function of the individual stress response across groups, we included aversiveness ratings of the SECPT and individual cortisol reactivity (T2–T3) as covariates in separate second‐level GLM analyses on NOW versus LATER contrasts.

#### 2.7.2 DD

We used log‐transformed *k*‐values as the covariate in second‐level analyses on NOW versus LATER contrasts using the entire sample (SG and CG).

#### 2.7.3 Cue‐Type Effects

We examined cue‐type effects in two steps. First, within the CG, first‐level t‐contrasts for each cue type separately (NOW > LATER and LATER > NOW for food and cigarette cues) were submitted to second‐level one‐sample *t*‐tests. Second, to examine the influence of stress exposure on cue‐type effects, we used full‐factorial models with the factors cue type (food, cigarette) and group (SG, CG) for each strategy contrast (NOW > LATER and LATER > NOW).

## 3 Results

### 3.1 Task Effect

#### 3.1.1 Behavioural

We found lower subjective craving for LATER compared to NOW, *t*(34) = −10.14, *p* < 0.001., *d* = −1.71, indicating successful ROC.

#### 3.1.2 Neural

**FIGURE 2:**
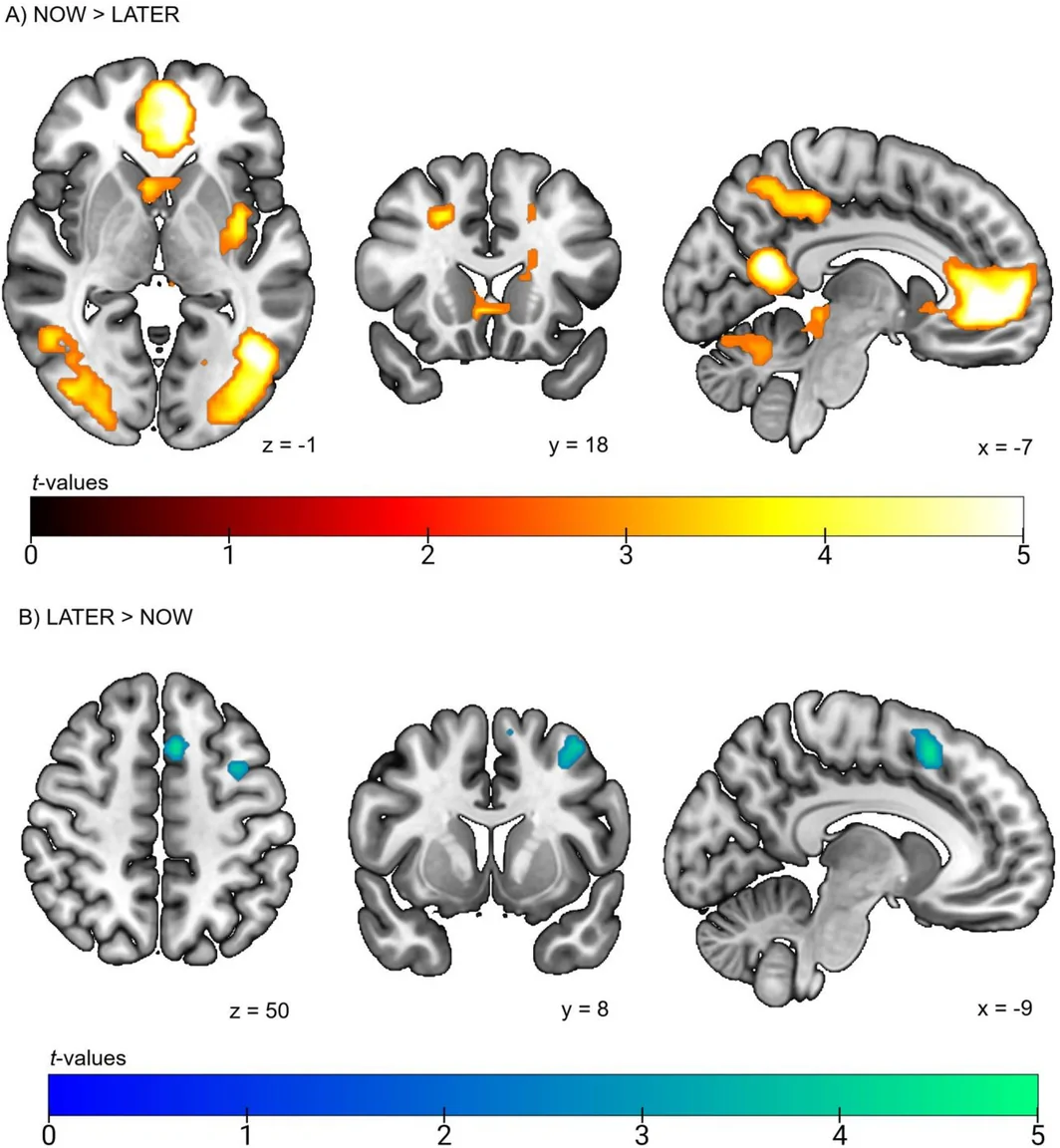
Task activation within CG during NOW versus LATER. (A) Activation in the NOW > LATER t‐contrast in the control group (CG) across both cue types. Image was created using a threshold of *p* = 0.005, FWE correction at cluster level. (B) Activation in the LATER > NOW t‐contrast in the control group (CG) and across both cue types. Image was created using a threshold of *p* = 0.005. All images were created with MRIcroGL using an MNI template. MNI, Montreal Neurological Institute.

We observed significantly lower activation in the VS/sgACC and the vmPFC/OFC ROIs and higher activation in the dlPFC ROI during LATER compared to NOW. The whole brain results further indicated higher activation in a broad network of regions, including the precuneus and mPFC during NOW compared to LATER, although no regions showed significant activation in the LATER > NOW contrast (see Table 2 and Figure 2). To explore a direct relationship between craving and brain activation, we performed a parametric regression using trial‐by‐trial craving ratings. The results revealed a positive relationship between subjective craving and activity in the VS/sgACC (peak *x, y, z* = −6, 14, −4, *t* = 7.06, *p*_*FWE*_ < 0.001) and vmPFC/OFC (peak *x, y, z* = −9, 53, 2, *t* = 9.56, *p*_*FWE*_ < 0.001; see Section S3.5).

**TABLE 2:** Results for fMRI analysis.

	MNI coordinates			Statistical values		
	x	y	z	*k*	*t*	*p*_*FWE*_
Effect of strategies within CG						
NOW > LATER						
Whole brain level						
Superior frontal gyrus, dorsolateral L	−15	29	38	28	7.15	0.001
Anterior cingulate cortex, pregenual L	−6	41	2	26	6.30	0.006
Precuneus_L	−3	−61	17	9	6.11	0.010
Superior frontal gyrus, medial L	−9	56	5	5	5.73	0.026
Region of interest^a^						
vmPFC/OFC	−6	47	−4	257	5.79	< 0.001
VS/sgACC	3	14	−4	11	4.33	0.012
LATER > NOW						
Region of interest^a^						
dlPFC	−39	5	56	22	4.09	0.038
Linear effect of stress						
NOW > LATER						
Whole brain level						
Middle orbital gyrus^b^ L	−27	41	−7	57	5.99	0.001
Middle frontal gyrus R	39	29	32	13	5.55	0.005
IFG pars orbitalis L	−42	20	−4	7	5.45	0.007
Superior frontal gyrus, medial L	−9	59	2	8	5.33	0.011
Region of interest^a^						
vmPFC/OFC	−9	59	2	194	5.33	< 0.001
Linear effect of delay discounting						
NOW > LATER						
Region of interest^a^						
vmPFC/OFC	−12	56	8	9	3.58	0.036

*Note:* Peak level: *t*, test statistic; *p*, significance value with FWE correction; L, left hemisphere, R, right hemisphere. Labelling for results at the whole brain level was done with AAL3; only significant results for k ≥ 5 are shown.

Abbreviations: CG, control group; k, cluster size in voxels; MNI, Montreal Neurological Institute; SG, stress group.

^a^ Region of interest. Using a combined mask with small volume correction with familywise error corrected at *p* = 0.05 with a voxelwise threshold of *p* = 0.005.

^b^ Labelling was done with Anatomy Toolbox as it was not available in AAL3.

### 3.2 Stress Effect

#### 3.2.1 Manipulation Check

**FIGURE 3:**
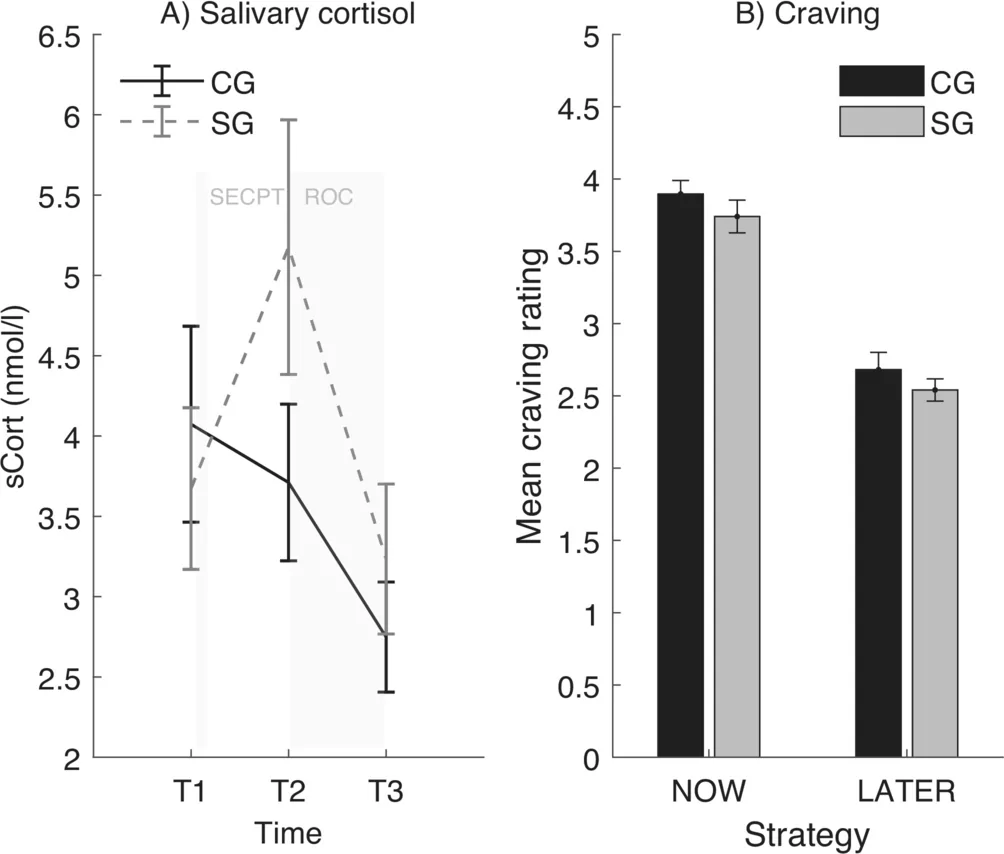
Stress effects on salivary cortisol and regulation of craving. (A) The trajectories of raw salivary cortisol from before the stress induction (T1), 18 min after the stress induction (T2) and after completion of the ROC task (T3) are presented per group. The times of stress induction (SECPT, socially evaluated cold pressor task) and ROC task (ROC, regulation of craving) are shown in green. (B) Craving per strategy and group. Error bars reflect standardized error of the mean. CG, control group; sCort (nmol/L), salivary cortisol (nanomole per litre); SG, stress group.

The SG reported higher aversiveness ratings (*M* = 3.6, SD = 1.1) than the CG (*M* = 1.6, SD = 0.7), indicating successful stress induction, *t*(54.01) = 8.40, *p* < 0.001, *d* = 2.06. Cortisol levels did not differ between groups at T1 and T3 (all *ps*_*uncorr*_ > 0.45), but cortisol reactivity (T2–T1) was higher in SG than CG, *t*(42.29) = 3.55, *p*_*BonfHolm*_ = 0.004, *d* = 0.87 (see Figure 3A). A similar difference emerged during the ROC task (T2–T3), *t*(66) = 2.06, *p*_*uncorr*_ = 0.044, *d* = 0.50, but this did not survive correction for multiple comparison (*p*_*BonfHolm*_ = 0.131).

#### 3.2.2 Behavioural

The mixed‐model ANOVA on subjective craving (see Figure 3B) resulted in a significant main effect of strategy, *F*(1, 66) = 212.96, *p* < 0.001, η^2^_p_ = 0.76, indicating successful ROC in both groups, with lower craving for LATER compared to NOW (*t*(67) = −14.71, *p* < 0.001, *d* = −1.78). However, neither the main effect of group, *F*(1, 66) = 1.54, *p* = 0.218, nor the interaction between group and strategy, *F*(1, 66) = 0.01, *p* = 0.926, reached significance. Regression analyses indicated that neither subjective stress effects in the SECPT (i.e., aversiveness ratings), nor cortisol levels were related to ROC success, all *ps*_*uncor*_ > 0.459.

#### 3.2.3 Neural

**FIGURE 4:**
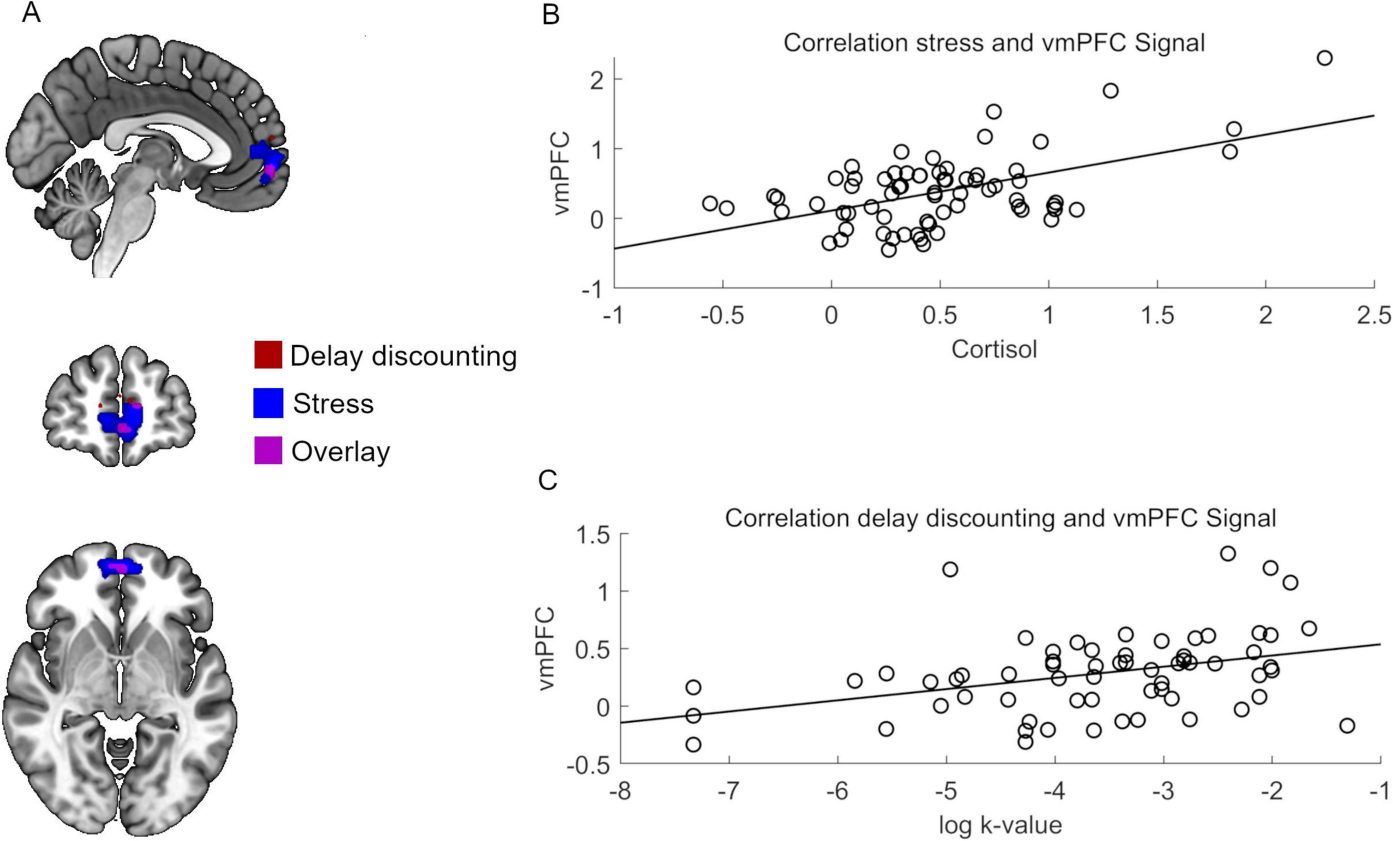
Effect of stress and DD on NOW > LATER*.* (A) Effect of stress (cortisol T2–T3) and DD (log *k*‐value) on the NOW > LATER contrast within the vmPFC/OFC ROI across both cue types. A threshold of *p* = 0.005 and vmPFC/OFC ROI mask was used, with MRIcroGL using an MNI template (MNI, Montreal Neurological Institute). (B) Scatter plot of relationships between stress (cortisol T2–T3) and mean contrast estimates in the NOW > LATER contrast in a 6‐mm sphere around the activation peak [−9, 59, 2]. (C) Scatter plot of relationships between DD (log *k*‐value) and mean contrast NOW > LATER in a 6‐mm sphere around the activation peak [−12, 56, 8].

Similar to behavioural results, the comparison of groups on LATER > NOW and NOW > LATER contrasts did not indicate significant differences. Although aversiveness was not related to differential ROC‐related brain activation, we found that individual cortisol levels were positively associated with higher brain responses in the vmPFC/OFC ROI (NOW > LATER), but not in other ROIs or whole‐brain analyses (see Table 2 and Figure 4).

### 3.3 DD

#### 3.3.1 Behavioural

Log‐transformed *k*‐value did not predict subjective ROC success, *F*(1, 61) = 0.68, *p* = 0.413. Exploration of a possible interaction between DD and cortisol on ROC success did not reveal a significant result, *p* = 0.876.

#### 3.3.2 Neural

Log‐transformed *k*‐value had a significant positive relationship with higher brain responses in the vmPFC/OFC (NOW > LATER; see Table 2 and Figure 4), but not in other ROIs or whole‐brain analyses.

Figure 4 shows the overlapping effects of cortisol reactivity and DD *k*‐value. We explored a possible interaction and included both factors as covariates in a second level, which neither showed significant results at the whole brain level nor in the craving circuit ROI, indicating that the separate effects are small.

### 3.4 Cue‐Type Specific Effects

#### 3.4.1 Behavioural

The mixed‐model ANOVA on subjective craving (see Figure 3) yielded a significant strategy × cue‐type interaction, *F*(1, 66) = 6.55, *p* = 0.013, $\eta_{p}^{2}$ = 0.09, whereas the main effect of cue type did not reach significance, *F*(1, 66) = 1.30, *p* = 0.259. Post hoc *t*‐tests revealed successful ROC for both cue types, with lower craving ratings for LATER‐drug compared to NOW‐drug, *t*(67) = −14.22, *p*_*BonfHolm*_ < 0.001, *d* = −1.72, and LATER‐food compared to NOW‐food, *t*(67) = −13.36, *p*_*BonfHolm*_ < 0.001, *d* = −1.62. Regulation success was higher for drug cues compared to food cues *t*(67) = 2.57, *p*_*BonfHolm*_ = 0.038, *d* = 0.31. However, separate comparisons of craving ratings for each cue type within each strategy did not reveal any differences (both *ps* > 0.12).

#### 3.4.2 Neural

When analysing cue types separately, we observed strategy effects for cigarette cues, with greater activation for NOW‐drug than LATER‐drug in precuneus, ACC and the VS and vmPFC ROIs. For food cues, we observed a strategy effect in the vmPFC ROI, with greater activation for NOW‐food than LATER‐food. No significant effects were found for the reverse contrast, LATER > NOW, likely due to reduced statistical power in the cue‐type specific analyses. In the full‐factorial models including cue type and group as factors, we found a main effect of cue type in the precuneus for NOW > LATER contrast. Specifically, activation was greater for cigarette cues than for food cues, with peaks at *x, y, z* = 9, −55, 17, *F* = 34.46, *p* = 0.001 and *x, y, z* = −9, −58, 17, *F* = 27.83, *p* = 0.003, both FWE corrected at peak level (see Table 3). We found neither a main effect of group nor a group × cue‐type interaction.

**TABLE 3:** Results for fMRI analysis.

	MNI coordinates			Statistical values		
	x	y	z	*k*	*t*	*p*_*FWE*_
Effect of strategies within CG						
NOW‐drug > LATER‐drug						
Whole brain level						
Precuneus_L	−9	−58	17	63	7.08	0.001
Anterior cingulate cortex, pregenual L	−6	41	−1	33	6.74	0.002
Superior frontal gyrus, dorsolateral L	−21	32	38	31	6.43	0.004
Region of interest^a^						
vmPFC/OFC	−6	47	−4	248	5.73	> 0.001
VS/sgACC	0	17	−4	7	4.03	0.022
LATER‐drug > NOW‐drug						
No suprathreshhold clusters	—	—	—	—	—	—
NOW‐food > LATER‐food						
No suprathreshhold clusters	—	—	—	—	—	—
Region of interest^a^						
vmPFC/OFC	−6	59	5	102	4.19	0.18
LATER‐food > NOW‐food						
No suprathreshhold clusters	—	—	—	—	—	—
Region of interest^a^						
dlPFC	−36	5	53	26	4.12	0.037
Effects of group and cue‐type on NOW > LATER						
SG > CG						
No suprathreshhold clusters	—	—	—	—	—	—
CG > SG						
No suprathreshhold clusters	—	—	—	—	—	—
Cigarettes > Food						
Whole brain level						
Precuneus_R	9	−55	17	30	5.87	< 0.001
Precuneus_L	−9	−58	17	20	5.27	0.005
Precuneus_L	−12	−46	8	5	4.85	0.022
Middle occipital gyrus R	45	−64	26	5	4.73	0.034
Region of interest^a^						
vmPFC/OFC	−3	47	−4	26	3.40	0.044
Food > Cigarettes						
No suprathreshhold clusters	—	—	—	—	—	—

*Note:* Peak level: *t*, test statistic; *p*, probability value with FWE correction; L, left hemisphere, R, right hemisphere; n/a, no label found. Labelling was done with AAL3.

Abbreviations: CG, control group; k, cluster size in voxels; MNI, Montreal Neurological Institute; SG, stress group.

^a^ Region of interest. Using a combined mask with small volume correction with familywise error corrected at p = 0.05 with a voxelwise threshold of p = 0.005.

## 4 Discussion

The aim of the present study was to explore the role of stress and DD during ROC in smokers. As expected, considering long‐term negative consequences (i.e., LATER strategy) was associated with lower subjective craving accompanied by lower activation in the craving network (i.e., VS, vmPFC/OFC, anterior cingulate cortex [ACC]), extending to regions associated with the default‐mode network (i.e., the precuneus) and higher activation in the dlPFC. We did not observe differences in these networks and subjective craving ratings between stress and control groups; however, individual cortisol reactivity and DD were positively associated with differential brain activation in the vmPFC across the entire sample.

### 4.1 Task Effect

The observation that LATER is associated with lower craving and activation in the VS/sgACC and vmPFC/OFC, and higher activation in the dlPFC is consistent with previous findings [8, 11]. As VS and vmPFC mediate subjective decision values [4], this suggests that successful ROC may attenuate the subjective value of a desired option. This could facilitate reduced use, as brief ROC trainings lowered the amount of snack intake [10] and cigarettes smoked per day [38]. We could replicate higher activation of the prefrontal control network during LATER in dlPFC, but not vlPFC [8, 11]. Although the location and magnitude of the effect were consistent with previous studies [8, 11, 12], the effect appeared small compared to the differences in activation of craving‐related areas. One reason for this rather small effect is that participants used imagination in both strategies. As a result, performing the NOW strategy may have also engaged prefrontal control, as we found activation in superior frontal areas. For instance, a ROC study in cocaine users [9] indicated that the NOW strategy involved up‐regulation of craving relative to a control condition (naturally attending to drug and food cues). Further, a tDCS study found impaired execution of both NOW and LATER when the dlPFC was deactivated [39].

An involvement of the precuneus in ROC is consistent with previous reports [8, 11]. The precuneus is a central hub of the DMN [40], which is associated with both passive states [41] and active, internal states such as self‐referential and future‐oriented thinking [26]. A recent line of research links the DMN to imagining the future self, where activation of the precuneus is associated with vividness [42] and that of the vmPFC with valence [42]. Similar to our results, this line of research also found stronger activation of the striatum during imagination with positive valence [42]. Given the prospective nature of ROC strategies, our finding that the precuneus showed greater activation in response to cigarette cues for NOW > LATER contrast may suggest, particularly for smoking‐related cues, the NOW strategy involves especially vivid, emotionally engaging and positive mental representations of the future self (e.g., enjoying a cigarette after participating in the study). Notably, this neural cue‐type effect was not accompanied by corresponding differences in consciously perceived craving between cigarette and food cues. In contrast, lower precuneus during LATER could indicate reduced self‐referential processing with regard to negative long‐term consequences, which might be challenging and aversive to relate to personally. Because the influence of prospection on decisions seems to depend on its emotional intensity [43], future research should investigate to what extent ROC success depends on the ability to vividly visualize consequences anticipated for oneself.

### 4.2 Stress

We found no group difference between the SG and CG in subjective craving ratings or neural activation in the ROC task. Given our assumption that stress might impair ROC success, this was counter to our hypotheses. However, because control‐related areas were less prominently involved than expected in the current study, it also follows that stress may have been less able to affect ROC performance via this pathway. Furthermore, this fits in with recent findings that confirm ROC training effects on snack intake even under stress [44]. In terms of therapeutic implications, focusing on long‐term negative consequences is therefore worth investigating as a tool that could prove effective even in stressful situations. However, although there was no group difference in neural activation during ROC, the individual cortisol level during the task was associated with greater differentiation of activity in craving‐related areas. We consider two explanations why stress affected areas that showed greater activation during NOW compared to LATER.

First, higher activation during NOW, potentially reflecting increased valuation of the immediate reward or stress relief tied to smoking, is consistent with evidence that acute stress increased anticipatory processing in reward circuits [45]. In turn, if more potent stressors turned out to be able to bring this relationship to bear on drug seeking, our result would emphasize the need to counteract this by implementing and training ROC under stress.

Second, stress reactivity could be tied to stronger vmPFC reduction during LATER, suggesting enhanced de‐valuation of smoking. Such facilitation of ROC, at least at the neural level, would be in line with the claim that intermediate stress levels can support cognitive functioning [46]. In sum, individual stress reactivity should be considered as a potential, context‐dependent modulator of smoking‐related value computations.

### 4.3 DD

DD was associated with higher differentiation of value‐related activation in the vmPFC between NOW and LATER. This is consistent with meta‐analytic evidence on DD emphasizing the prominence of mPFC in integrating immediate over delayed outcomes into subjective value [47]. Validating the construct, stronger discounting of future events would be accompanied by stronger vmPFC signals for the pleasant immediate effects of smoking during NOW than the delayed risks of smoking during LATER. Whereas DD did not affect ROC behaviourally, it remains to be determined whether the current subtle neural effects translate into altered ROC performance in individuals specifically selected for extreme levels of DD.

Lastly, the overlap of stress reactivity and DD effects on the vmPFC is noteworthy, potentially resulting from stress inducing a state mirroring the pronounced orientation towards the present [48]. Our results then show that the LATER strategy, when used need not be less effective due to a higher discounting of the future in advance. One reason for this could be that previous research has shown that DD can be modified by prospection [49].

### 4.4 Limitations

The ROC paradigm would benefit from a control condition with passive viewing to delineate specific effects of NOW and LATER. As SECPT is a moderate stressor [33], effects may be more pronounced with more impactful stressors. A limitation of the present study is that perceived stress was not assessed in a before‐after comparison. Such a design would have allowed a more accurate assessment of subjectively perceived stress as well as temporal dynamics of different timings of the stress components. Although the ROC task was scheduled at the time of the cortisol peak reflecting slower HPA axis activation [28], it is possible that more pronounced impairments in regulation occur immediately after the stressor, mediated by the sympathetic nervous system activity rather than by HPA effects [50]. It should be mentioned that all participants were male and the self‐assessment of the test participants in the FTND was rather low on average. This prevents a generalization of our findings to females and severely addicted individuals.

### 4.5 Conclusion

This is the first study to show that smokers can maintain ROC performance and its neural correlates in the face of moderate stress. As prefrontal control seems to have played a limited role during ROC, smokers may have benefited from the involvement of the default mode network. Further, vmPFC signals differed both as a function of individual cortisol response and future‐oriented decision tendencies (i.e., DD), suggesting state‐ and trait‐dependent influences on integrating time into smoking‐related computations of value. In sum, while more challenging circumstances remain to be tested, our results suggest that ROC may prove a resilient intervention that could be especially robust if training schemes emphasized self‐referential future thinking.

## Author Contributions

Solvej Nickel‐Grenke and Raoul Wüllhorst share the first authorship. Raoul Wüllhorst and Tanja Endrass were responsible for the study concept and design. Solvej Nickel‐Grenke and Raoul Wüllhorst contributed to the acquisition of the data. Solvej Nickel‐Grenke performed the analysis and provided the original draft for the manuscript. Stefan Scherbaum provided code of the DD task and scripts for the analysis. Kristina Schwarz, Raoul Wüllhorst and Tanja Endrass assisted with data analysis, interpretation of findings and provided critical revision of the manuscript. All authors critically reviewed content and approved the final version for publication.

## Funding

This work was supported by the Deutsche Forschungsgemeinschaft (DFG, grant number EN 906/6‐1).

## Ethics Statement

The local ethics committee approved the study protocol (reference number EK 53022018).

## Consent

Participants provided informed consent in accordance with the Declaration of Helsinki.

## Conflicts of Interest

The authors declare no conflicts of interest.

## Supporting information


**Table S1:** Exploratory results for the effect of task in the entire sample pooled across both picture types when using separate regressors for strategy cue and picture.
**Table S2:** Results for fMRI analysis.

## Data Availability

The data that support the findings of this study are available on request from the corresponding author. The data are not publicly available due to privacy restrictions. The second‐level t maps and task data are made publicly available on https://osf.io/ydnzj/files/osfstorage.
