# Supplementary material for: Regulation of Craving: Neural Correlates and the Role of Stress and Delay Discounting
Source: Addict Biol. 2026 Sep 24;31(10):e70190. doi: 10.1111/adb.70190 (PMC13612162; doi:10.1111/adb.70190)
Supplement: Supplementary file 1 [file ADB-31-e70190-s001.docx]

**Supplementary Information**

This supplement contains relevant additional information referenced in the paper. In order to document any deviations from the preregistration of this project, we quote sections from the preregistration, supplemented by paragraph indicating relevant changes. Please note that the results presented in this paper refer to two preregistrations (which were created and published at the same time), as they were planned as two separate papers. However, due to unexpected results, they were combined into one paper to ensure consistent presentation of all results. This supplement is divided into three main components: Additional information as a supplement to the main text (S1), presentation of deviations from the preregistration (S2), results of preregistered analyses not listed in the main text (S3).

**S1 Additional information**

S1.1 Sample

S1.1.1 Sample Size Estimation

“Sample size estimation was conducted for analysis of variance with power (1 - β) = .80 and α = .05 using G*Power 3(1), focusing on the group (stress, control) by regulation (NOW, LATER) interaction. Assuming η^2^_p_ = .06 for this effect based on a previous study on stress impacting emotion regulation (2), the necessary sample size would be n = 34. Regarding the interaction of salivary responses with executive functioning in the regression model predicting regulatory capacity, we used the powersim function implemented in Stata Statistical Software (StataCorp LLC, College Station, TX) for power analysis. We entered a standardized beta coefficient of β = 0.42 based on prior work examining the moderating effects of working memory capacity on the relationship between sCort response and model-based behavior (2). Further inputs were β = 0.35 and β = -0.25 for the main effects of executive functioning (3) and saliva measures (2), respectively, in simulations with 1000 repetitions. Even with Bonferroni-corrected α = .050 / 3 = .0167 (three measures of executive functioning will be obtained), simulations still suggested sufficient power (1 - b) = .82 to detect the interaction with n = 60 subjects.”

(Pages 4f, <https://osf.io/dgs7r>)

S1.1.2 Reasons for data exclusion during analysis

We had to exclude n = 6 participants due to dropout before the second appointment; n = 14 participants had to be excluded due to insufficient quality of fMRI data (n = 3 with incidental medical findings, n = 3 with missing data in VS ROI [< 80 %], n = 1 with missing field map due to technical problems, n = 7 due to excessive movement [> 3mm in more than 30% of all trials]).

S1.1.3 Exclusion criteria during data collection

Exclusion criteria were (1) current attempt to quit smoking to ensure homogeneously appetitive motivation toward smoking cues; (2) depression or anxiety disorders, psychoactive medication, psychological or psychiatric treatment, or use of illicit drugs other than cannabis within the past three months; (3) consistent vegan or vegetarian diet or current diet to lose weight; (4) left-handedness; (5) hormone intake; (6) food allergies causing high-caloric foods to be perceived as aversive; (7) work in night shifts one week prior to testing; (8) drinking more than 15 standard drinks per week on a regular basis in the past twelve months; (9) disagreeing to abstain from cannabis one week prior to testing; (9) fMRI contraindications; (10) past or present neurological disorder, cardiovascular disease, head trauma, or other mental disorders than mentioned above.

S1.2 Additional tasks

The following tasks were conducted outside of the MRI scanner during a purely behavioral session (~1h), which took place within one week before the scanning session.

S1.2.1 Stop signal task (SST)

A white circle on a gray background remained empty for 300-500 ms. Then a green right- or left-pointing arrow appeared in its center for 1000 ms, asking to respond according to its direction as quickly and accurately as possible with the index fingers. On stop trials (25%), the arrow turned red after a certain interval (stop signal delay, SSD), asking to cancel the already initiated response. SSDs increased or decreased by 50 ms (lower and upper limits: 50 and 350 ms) after successful or failed inhibitions, respectively. The task started with four SSDs (100, 150, 200, and 250 ms), each active on 25% of stop trials and tracked by its own adaptation algorithm (4). Go and stop trials were presented in pseudorandom order with the following constraints: The first three trials were go trials, left and right arrows occurred equally often within go and stop trials, stop trials and arrow direction did not repeat more than twice. The inter-trial interval was 400-800 ms. The task stopped after two valid blocks (stopping accuracies between 40 and 60%) or a maximum of four blocks (128 trials each). Invalid blocks were not analyzed, and all participants included in the analyses completed at least two valid blocks. We computed individual stop-signal reaction time using the integration method as per recent recommendations (5). All participants included in the analyses fulfilled the prerequisite of two valid blocks and that the mean response time on failed stop trials was smaller than on go trials.

S1.2.2 Working memory task

Each trial started with an intertrial interval (ITI) of 1000 ms, featuring a white cross in the center. Then, participants viewed nine squares (white outline, empty interior) on a black background and a subset of squares (>= 3) consecutively “lit up” (interior turned white) for 1000 ms while participants were asked to watch and memorize that sequence. After another 1000 ms, participants again viewed the nine empty squares and were instructed to reproduce the previous sequence in reverse order, beginning with the last and ending with the first, using the computer mouse. The interior of a square turned white while the cursor of the mouse hovered over it and upon clicking on a square, the interior remained white, and the outline turned red. Selections could not be reversed and there was no time limit for responding. After another 1000 ms, participants viewed feedback whether they had responded accurately (German words for “Correct” or “Wrong”) for 1000 ms. The task started with a sequence length (working memory load) of n = 3. If participants correctly reproduced a sequence, working memory load increased by one on the next trial. If they reproduced a sequence incorrectly, the next trial featured a different sequence but working memory load remained unchanged. If participants responded incorrectly a second time for that working memory load, the task terminated. We quantified working memory capacity as the maximum working memory load for which participants could correctly reproduce the sequence in reverse order.

S1.3 fMRI data acquisition and analysis

“Participants were scanned at the Neuroimaging Center, TUD. Due to a planned scanner upgrade we had to continue data collection (n=74 surveyed before with SIEMENS, 3T, MAGNETOM TrioTim syngo MR B17) with slightly different settings.

| MRI system | SIEMENS, 3T, MAGNETOM TrioTim syngo MR B17 | SIEMENS MAGNETOM 3.0T XR Numaris/X VA30A-03DZ |
| --- | --- | --- |
| Pulse sequence | Gradient echo | Gradient echo |
| Image type | Structural scan: 3D-MPRAGE  Functional scans: EPI | Structural scan: 3D-MPRAGE  Functional scans: EPI |
| Essential sequence & imaging parameters | | |
| T1-weighted anatomical images | TR 2400 ms  TE 2.19 ms  Flip angle 8°  Acquisition time 7:02 min  Field of View 272 mm  Field of View Phase 100.0%  Voxel size 0.85 mm³  Number of Slices 240  Slice Orientation S > T-2.1 > C-0.6 ascending | TR 2400 ms  TE 2.20 ms  Flip angle 8°  Acquisition time 7:02 min  Field of View 272 mm  Field of View Phase 100.0%  Voxel size 0.85 mm³  Number of Slices 240  Slice Orientation sagittal ascending |
| Functional MRI ROC-task | TR 1880 ms  TE 25 ms  Flip angle 76°  Acquisition time 31:26 min  Field of View 220 mm  Field of View Phase 100.0%  Voxel size 3.4 x 3.4 x 3.0 mm  Number of Slices 38  Slice Orientation T > C-18.2 > S0.7; sequential, ascending  Phase Orientation A >> P | TR 1880 ms  TE 25 ms  Flip angle 76°  Acquisition time 31:26 min  Field of View 220 mm  Field of View Phase 100.0%  Voxel size 3.4 x 3.4 x 3.0 mm  Number of Slices 38  Slice Orientation Transversal sequential ascending  Phase Orientation A >> P |
| Parallel imaging method & parameters | None | None |
| Multiband parameters | None | None |
| Brain coverage | Whole-brain if it fits into field of view, else focus on full coverage of frontal and temporal areas | Whole-brain if it fits into field of view, else focus on full coverage of frontal and temporal areas |
| Field maps  Subsequent to functional scan, a field map will be recorded to control later on for spatial distortions. | Essential parameters of the fieldmap for further analyses are: TR = 439 ms, TE1 = 5.32 ms, TE2 = 7.78 ms; number of slices = 40, voxel size = 3.4 x 3.4 x 3.0 mm | Essential parameters of the fieldmap for further analyses are: TR = 439 ms, TE1 = 5.32 ms, TE2 = 7.78 ms; number of slices = 40, voxel size = 3.4 x 3.4 x 3.0 mm |
| Fat suppression | for the anatomical scan water stimulating, fast, for functional MRI ROC-task fat saturated. | or the anatomical scan water stimulating, fast, for functional MRI ROC-task fat saturated. |

“

(Pages 8ff, <https://osf.io/dgs7r>)

**S2 Deviations from the preregistration**

S2.1 Preprocessing

S2.1.1 Preprocessing information from preregistration

- “Estimation of a voxel displacement map using the fieldmap
- Realignment of the functional images to the mean image
- Slice Timing correction (reference to the middle slice in time) using SPMs 12’s Fourier transform
- Segmentation of the coregistered structural image
- Normalization to the MNI standard brain
- Smoothing of the normalized functional scans to reduce noise”

(Page 4 <https://osf.io/4wh3u> and page 5 <https://osf.io/ypwhr>)

S2.1.2 Preprocessing – changes to preregistration

To account for the small differences before and after the scanner upgrade, the data were preprocessed separately (the total readout time [used in the determination of the voxel displacement map] differs: before the upgrade 18.2701ms, after the upgrade 17.6399s). To account for possible effects of the scanner upgrade, we included scanner type as an additional regressor of no interest in all second-level analyses.

To specify:

- The realignment is based on the following parameters: a 6-parameter rigid body Transformation, 4th degree B-Spline Interpolation, least square method, unwarping B0 distortions with the voxel displacement map.
- Smoothing was done using a 8mm FHWM Gaussian Kernel

S2.2 First Level

S2.2.1 First Level - information from the preregistration

“[…]as well as the rating period, duration of abstinence and six movement parameters as regressors of no interest.”

(Page 5 <https://osf.io/ypwhr>)

“We do not intend to separate the instruction cue period from the picture presentation as they have been shown to be highly correlated (6), but we will explore whether this is also true in our sample.”

(Page 5 <https://osf.io/4wh3u>)

S2.2.2 First level - changes to preregistration

Only the rating period and movement parameters were used as regressors of no interest. As preregistered, we examined the results when separate regressors for strategy cue and picture presentation time were used in the first level. The results can be found in Table T2.

S2.3 Second Level

S2.3.1 Second Level – information from preregistration

“ROIs will be based on peaks (Talairach coordinates) found in prior work (6, 7). We will use spherical ROIs (6mm) around VS/sgACC (-3 11 -2) and vmPFC/OFC (0 56 -2) for the craving circuit and around dlPFC (-36 -1 55) and vlPFC (-45, 23, -8) for the cognitive control network. We transform the coordinates in MNI space. […] We will use FDR correction.”

(Page 5 <https://osf.io/4wh3u>)

“We repeat all analyses at a whole brain level (at a threshold of *p* < .001, uncorrected) and for food pictures. “

(Page 6 <https://osf.io/ypwhr>)

S2.3.2 Second level – changes to preregistration

To account for possible effects of the scanner upgrade, we included this an additional regressor of no interest (scanner type) in all second-level analyses. We decided to report FWE corrected results. As FWE is more frequently used, this helped us to better situate the analyses within the context of prior results. See Table T1 for effects of picture type. To account for the size of the structures, we have decided to extend the radius to 15mm for vmPFC/OFC, dlPFC, and vlPFC.

**S3 Results of preregistered analyses not listed in the main text**

S3.1 Analysis of craving

S3.1.1 Analysis of craving - information from the preregistration

“We will compare one-item craving ratings before and after the ROC task using a mixed-model ANOVA with the within-subjects factor time (pre-task, post-task) and the between-subjects factor group (control, stress). Furthermore, we will compare craving (QSU) before and after stress induction using a mixed-model ANOVA with the within-subjects factor time (pre-stress, pre-task, post-task) and the between-subjects factor group (control, stress).”

(Page 5 <https://osf.io/4wh3u>)

S3.1.1 Analysis of craving - results

The mixed-model ANOVA with the factors time (pre-task, post-task) and additional within-subjects factor cue-type (cigarette, food) and between subjects factor group (stress, control) on one-item craving ratings revealed a significant main effect of time, *F*(1, 66) = 29.15, *p* < .001, η^2^_p_ = 0.31, indicating that subjective craving increased from pre- to post-task (cigarettes: *t*(67) = 2.85, *p_ucnorr_* < .01, *d* = 0.35; food: *t*(67) = 5.67, *p_ucnorr_* < .001, *d* = 0.69). Furthermore, we found a significant time*cue-type interaction *F*(1, 66) = 12.41, *p* < .001, η^2^_p_ = 0.16, indicating higher craving for cigarettes compared to food pre-task, *t*(67) = 2.85, *p_ucnorr_* < .01, *d* = 0.35, but no difference post-task, *t*(67) = 0.36, *p_ucnorr_* = .720. There were neither main effects of group and cue-type nor interactions with group (all *ps* > .220).

With regard to evaluating the QSU/FCQ, there were 12 missing values. As this variable is not relevant for the hypotheses, we refrained from analysing these low-quality data**.**

S3.2 Analysis of stress

S3.2.1 Analysis of stress – information from preregistration

“We set up regression models with sAA as the predictor and run it for each dependent variable (subjective regulation success, LATER>NOW contrast, NOW>LATER contrast) to test exploratively for an influence of sAA.”

(Page 6 <https://osf.io/ypwhr>)

S3.2.2 Analysis of stress – results

We log-transformed alpha-amylase values to account for skewness. Regarding alpha-amylase, no significant differences between groups were found (all *ps_uncor_* > .428). Alpha-amylase level (T2-T3) was not related to ROC success, p = .820 and NOW vs. LATER contrast at the whole-brain-level FWE corrected.

S3.3 Analysis of executive functions

S3.3.1 Analysis of executive functions – information from preregistration

“We will try to form a latent factor EF from working memory, delay discounting and response inhibition. If successful, we will use it in the confirmatory analyses and only test the individual EFs (WM, DD, SSRT) as predictors on an exploratory basis.”

(Page 6 <https://osf.io/ypwhr>)

S3.3.2 Analysis of executive functions – results

There was no correlation between the three variables, all *p*s > .240, so a latent factor was not formed and they were analysed separately. Shorter SSRT was related to higher ROC success, b = -.262, *t*(66) = -2.20, *p_uncorr_* = .031. Adding cortisol reactivity, *p_uncorr_* = .745, and its interaction with SSRT, *p_uncorr_* = .700, to the model removed the SSRT effect, *p_uncorr_* = .099. Neither WM backward span, p_uncorr_ = .479, nor DD, *p_uncorr_* = .413, were related to ROC success. WM and DD also did not interact with cortisol reactivity to predict ROC success, *ps_uncorr_* > .770.

S3.4 Results for analysis with separate regressors for instruction and picture presentation

| *Table T1.* Exploratory results for the effect of task in the entire sample pooled across both picture types when using separate regressors for strategy cue and picture | | | | | | |
| --- | --- | --- | --- | --- | --- | --- |
|  | MNI Coordinates | | | Statistical values | | |
|  | x | y | z | *k* | *t* | *p_FWE_* |
| NOW > LATER Picture | | | | | | |
| Precuneus_L | -9 | -58 | 17 | 224 | 8.31 | < .001 |
| Anterior cingulate cortex, pregenual L | -9 | 47 | -1 | 457 | 7.75 | < .001 |
| Lingual gyrus R | 15 | -85 | -10 | 364 | 6.81 | < .001 |
| Middle cingulate & paracingulate gyri R | 3 | -40 | 41 | 71 | 6.64 | < .001 |
| Inferior temporal gyrus L | -48 | -67 | -7 | 150 | 6.61 | < .001 |
| Superior frontal gyrus, dorsolateral R | 18 | 26 | 44 | 105 | 6.57 | < .001 |
| Postcentral gyrus L | -45 | -34 | 59 | 147 | 6.39 | < .001 |
| Superior frontal gyrus, dorsolateral L | -21 | 26 | 41 | 48 | 6.22 | .001 |
| Middle occipital gyrus L | -27 | -85 | 29 | 125 | 6.22 | .001 |
| Superior parietal gyrus R | 27 | -58 | 47 | 104 | 6.20 | .001 |
| Rolandic operculum L | -39 | -4 | 14 | 22 | 6.17 | .001 |
| Superior occipital gyrus L | -9 | -97 | 8 | 54 | 5.74 | .003 |
| n/a | 0 | 17 | -4 | 8 | 5.72 | .004 |
| Middle cingulate & paracingulate gyri R | 3 | 8 | 29 | 13 | 5.61 | .005 |
| SupraMarginal gyrus L | -60 | -28 | 41 | 14 | 5.53 | .007 |
| Inferior temporal gyrus R | 54 | -52 | -4 | 11 | 5.42 | .010 |
| Superior parietal gyrus L | -21 | -61 | 47 | 13 | 5.41 | .010 |
| Middle frontal gyrus R | 45 | 41 | 8 | 16 | 5.38 | .011 |
| Fusiform gyrus L | -27 | -46 | -16 | 1 | 5.02 | .036 |
| Fusiform gyrus L | -30 | -70 | -13 | 3 | 5.00 | .038 |
| Caudate nucleus L | -18 | 8 | 17 | 1 | 4.95 | .044 |
| n/a | 21 | 23 | 26 | 1 | 4.91 | .047 |
| LATER > NOW Picture | | | | | | |
| Middle frontal gyrus L | -42 | 8 | 56 | 14 | 5.96 | .002 |
| Supplementary motor area L | -6 | 17 | 50 | 2 | 4.99 | .039 |
| NOW > LATER Strategy Cue | | | | | | |
| IFG pars orbitalis L | -33 | 26 | -7 | 6 | 5.29 | .013 |
| Crus II of cerebellar hemisphere R | 27 | -82 | -40 | 5 | 5.05 | .029 |
| LATER > NOW Strategy Cue | | | | | | |
| Middle occipital gyrus L | -12 | -94 | -1 | 60 | 9.14 | < .001 |
| Lingual gyrus R | 15 | -85 | -10 | 68 | 8.08 | < .001 |
| *Note.* MNI, Montreal Neurological Institute; k, cluster size in voxels; peak level: t, test-statistic; p, probability value with FWE correction; L, left hemisphere, R, right hemisphere. n/a, no label found; Labeling was done with AAL3. | | | | | | |

S3.5 Results for parametric regression

To directly investigate whether the regions identified in the NOW > LATER contrast were associated with subjective craving, we set up an exploratory first-level analysis using individual trial-by-trial craving ratings as parametric regressor. We then tested these effects at the second level across the whole brain and within the ROIs. The results (see Table T2) revealed a positive relationship between craving and activation in the ventral striatum, subgenual anterior cingulate cortex and the ventromedial prefrontal cortex, similar to those we found for a NOW>LATER contrast. We found no evidence of a negative association between subjective craving and brain activation.

| *Table T2.* Results for fMRI analysis | | | | | | |
| --- | --- | --- | --- | --- | --- | --- |
|  | MNI Coordinates | | | Statistical values | | |
|  | x | y | z | *k* | *t* | *p_FWE_* |
| Parametric Regression | | | | | | |
| Craving (positive relation) | | | | | | |
| Whole brain level | | | | | | |
| Calcarine fissure L | -6 | -94 | 8 | 173 | 10.24 | < .001 |
| Lingual gyrus R | 15 | -73 | -7 | 611 | 9.71 | < .001 |
| Anterior cingulate cortex, pregenual L | -9 | 53 | 2 | 984 | 9.56 | < .001 |
| Inferior temporal gyrus L | -48 | -67 | -10 | 268 | 9.12 | < .001 |
| Precentral Gyrus L | -36 | -25 | 56 | 265 | 8.09 | < .001 |
| Insula L | -39 | -1 | 11 | 53 | 6.75 | < .001 |
| Cuneus L | -12 | -58 | 20 | 95 | 6.36 | < .001 |
| Fusiform gyrus L | -33 | -43 | -13 | 30 | 6.05 | < .001 |
| Middle occipital gyrus L | -33 | -76 | 29 | 131 | 6.03 | < .001 |
| Middle frontal gyrus | -27 | 35 | 47 | 62 | 6.01 | < .001 |
| Middle cingulate & paracingulate gyrus R | 3 | -37 | 35 | 37 | 5.79 | < .001 |
| Caudate nucleus L | -15 | 11 | 20 | 17 | 5.75 | < .001 |
| Lobule VIII of cerebellar hemisphere | 6 | -64 | -31 | 20 | 5.63 | < .001 |
| Superior parietal gyrus | -15 | -61 | 47 | 15 | 5.34 | < .001 |
| Region of interest^a^ | | | | | | |
| vmPFC/OFC | -9 | 53 | 2 | 280 | 9.56 | < .001 |
| VS/sgACC | -6 | 14 | -4 | 11 | 7.06 | < .001 |
| Craving (negative relation) | | | | | | |
| Whole brain level | | | | | | |
| No suprathreshhold clusters | | | | | | |
| Region of interest^a^ | | | | | | |
| No suprathreshhold clusters |  |  |  |  |  |  |
| *Note*. MNI, Montreal Neurological Institute; k, cluster size in voxels; peak level: t, test-statistic; p, significance value with FWE correction; L, left hemisphere, R, right hemisphere; Labeling for results at the whole brain level was done with AAL3; only significant results for k ≥ 10 are shown.  ^a^Region of interest. Using a combined mask with small volume correction with familywise error corrected at *p* = .05. | | | | | | |

References

1.	Faul F, Erdfelder E, Lang AG, Buchner A (2007): G*Power 3: A flexible statistical power analysis program for the social, behavioral, and biomedical sciences. *Behav Res Methods*. 39:175-191.

2.	Otto AR, Raio CM, Chiang A, Phelps EA, Daw ND (2013): Working-memory capacity protects model-based learning from stress. *Proc Natl Acad Sci U S A*. 110:20941-20946.

3.	Schmeichel BJ, Demaree HA (2010): Working memory capacity and spontaneous emotion regulation: high capacity predicts self-enhancement in response to negative feedback. *Emotion*. 10:739-744.

4.	Aron AR, Poldrack RA (2006): Cortical and Subcortical Contributions to Stop Signal Response Inhibition: Role of the Subthalamic Nucleus. *Journal of Neuroscience*. 26:2424-2433.

5.	Verbruggen F, Aron AR, Band GPH, Beste C, Bissett PG, Brockett AT, et al. (2019): A consensus guide to capturing the ability to inhibit actions and impulsive behaviors in the stop-signal task. *eLife*. 8.

6.	Kober H, Mende-Siedlecki P, Kross EF, Weber J, Mischel W, Hart CL, et al. (2010): Prefrontal-striatal pathway underlies cognitive regulation of craving. *Proc Natl Acad Sci U S A*. 107:14811-14816.

7.	Suzuki S, Mell MM, O'Malley SS, Krystal JH, Anticevic A, Kober H (2020): Regulation of Craving and Negative Emotion in Alcohol Use Disorder. *Biol Psychiatry Cogn Neurosci Neuroimaging*. 5:239-250.
